# Ethopharmacological evaluation of antidepressant-like effect of serotonergic psychedelics in C57BL/6J male mice

**DOI:** 10.21203/rs.3.rs-3138705/v1

**Published:** 2023-07-07

**Authors:** Rika Takaba, Daisuke Ibi, Keisuke Yoshida, Eri Hosomi, Ririna Kawase, Hiroko Kitagawa, Hirotaka Goto, Mizuki Achiwa, Kento Mizutani, Kyosuke Maede, Javier González-Maeso, Shinji Kitagaki, Masayuki Hiramatsu

**Affiliations:** Meijo University; Meijo University; Meijo University; Meijo University; Meijo University; Meijo University; Meijo University; Meijo University; Meijo University; Meijo University; Virginia Commonwealth University School of Medicine; Meijo University; Meijo University

**Keywords:** serotonergic psychedelics, psilocin (psilocybin), DOI, TCB-2, major depressive disorder

## Abstract

Serotonergic psychedelics such as psilocybin, lysergic acid diethylamide, and DOI exert a hallucinatory effect through serotonin 5-HT 2A receptor (5-HT2A) activation. Recent studies have revealed that serotonergic psychedelics have therapeutic potential for neuropsychiatric disorders, including major depressive and anxiety-related disorders. However, the involvement of 5-HT2A in mediating the therapeutic effects of these drugs remains unclear. In this study, we ethopharmacologically analyzed the role of 5-HT2A in the occurrence of anxiolytic-and antidepressant-like effects of serotonergic psychedelics such as psilocin, an active metabolite of psilocybin, DOI, and TCB-2 in mice. Mice with acute intraperitoneal psychedelic treatment exhibited significantly shorter immobility times in the forced swimming test (FST) and tail-suspension test (TST) than vehicle-treated control mice 24 h post-treatment. These effects were eliminated by pretreatment with volinanserin, a 5-HT2A antagonist. Surprisingly, the decreasing immobility time in the FST in response to acute psilocin treatment was sustained for at least three weeks. In the novelty-suppressed feeding test (NSFT), the latency to feed, an indicator of anxiety-like behavior, was decreased by acute administration of psilocin; however, pretreatment with volinanserin did not diminish this effect. In contrast, DOI and TCB-2 did not affect the NSFT performance in mice. Furthermore, psilocin, DOI, and TCB-2 treatment did not affect the spontaneous locomotor activity or head-twitch response, a hallucination-like behavior in rodents. These results suggest that 5-HT2A contributes to the antidepressant effects of serotonergic psychedelics rather than an anxiolytic effects.

## 1. Introduction

Approximately 30–40% of patients with major depressive disorder (MDD) have treatment-resistant depression (TRD), defined as the failure of two or more medication trials, such as tricyclic antidepressants and selective serotonin reuptake inhibitors (SSRIs). Patients with TRD are more likely to attempt suicide than treatment responders (Nelsen and Dunner, 1995). Additionally, existing antidepressants require chronic dosing for several weeks, leading to an increased risk of adverse effects such as serotonin syndrome; i.e., altered mental status, autonomic hyperactivity, and neuromuscular abnormalities (Wisniewski et al., 2009, Foong et al., 2018). In the last 20 years, the *N*-methyl-D-aspartate (NMDA) receptor antagonist ketamine has shown rapid and sustained antidepressant effects in patients and animal models of depression (Berman et al., 2000, Zhang and Hashimoto, 2019). Further, Spravato^®^ (esketamine, the *S*-(+) enantiomer of ketamine) nasal spray was approved for the treatment of TRD by the U.S. Food and Drug Administration (FDA) as well as the European Commission (Turner, 2019, Singh et al., 2020). Unfortunately, esketamine has serious adverse effects, such as sedation and dissociation (Sapkota et al., 2021). Although 60–80% of patients with TRD are responsive to ketamine, the effects typically last for only 1–2 weeks at most (Ballard et al., 2018, Rong et al., 2018, Wilkinson et al., 2018). Therefore, safer and more effective therapeutic agents are required to treat patients with TRD. Carhart-Harris and colleagues have reported the rapid and lasting antidepressant effects of psilocybin, a hallucinogenic component of the psilocybe genus found in magic mushrooms (Hofmann et al., 1958), in patients with TRD (Carhart-Harris et al., 2016). Most patients with TRD had reduced depressive symptoms that lasted for at least three weeks (Carhart-Harris et al., 2018). These findings are supported by various clinical studies (Carhart-Harris et al., 2021, Davis et al., 2021, Goodwin et al., 2022, Gukasyan et al., 2022). In particular, a recent study revealed that psilocybin administration produces antidepressant effects in patients with MDD throughout a 12-month follow-up period (Gukasyan et al., 2022), indicating the incomparable long-term effects of psilocybin. Based on these reports, the FDA has approved psilocybin as a breakthrough medicine for clinical trials in MDD (Nutt et al., 2020).

Psilocybin is known as one of the “serotonergic psychedelics” that activate the cortical serotonin 5-HT_2_A receptor (5-HT2A), causing a hallucinatory effect (Gonzalez-Maeso et al., 2007, Nichols, 2016); however, involvement of 5-HT2A in the therapeutic effects of psilocybin remains unclear. Although, preclinical studies in mice have demonstrated the potential therapeutic effects of serotonergic psychedelics independent of 5-HT2A (Hesselgrave et al., 2021, Odland et al., 2021), other research groups have reported that 5-HT2A is required for the therapeutic effects of serotonergic psychedelics in rodents (Ripoll et al., 2006, Cameron et al., 2021, Cao et al., 2022). Therefore, it may be useful to investigate the antidepressant effects of various psychedelics with different affinities for 5-HT2A.

Serotonergic psychedelics are mainly classified into three structures: i.e., tryptamines such as psilocybin, ergolines such as lysergic acid diethylamide (LSD), and phenethylamines such as (±)-1-(2,5-dimethoxy-4-iodophenyl)-2-aminopropane (DOI), 2,5-dimethoxy-4-methylamphetamine (DOM), 2,5-dimethoxy-4-bromoamphetamine (DOB), and (4-bromo-3,6-dimethoxybenzocyclobuten-1-yl)methylamine (TCB-2) (McLean et al., 2006, Nichols, 2018). Although tryptamine and ergoline derivatives are nonselective 5-HT receptor agonists, phenethylamine derivatives exhibit higher selectivity for 5-HT2A than other structures (Nichols, 2012) (also see Table 1). In this study, we investigated the involvement of 5-HT2A in the behavioral modification by psychedelic tryptamines, such as psilocin, an active metabolite of psilocybin, and phenethylamines, such as DOI and TCB-2, using mice models. Additionally, we examined the effects of serotonergic psychedelics on spontaneous activity and hallucinatory-like behavior in mice. This study provides evidence for the use of 5-HT2A as a therapeutic target in MDD and related disorders.

## 2. Materials and methods

### 2.1. Animals

Male C57BL/6J mice (7–9 weeks old) were obtained from Japan SLC, Inc. (Hamamatsu, Japan). Unless otherwise stated, the mice were group-housed (4–5 mice/cage). The mice were kept in a well-regulated environment (24 ± 1°C, 55 ± 5% humidity) according to a 12-h light/dark cycle (lights on at 7:00 a.m.) and provided food and tap water ad libitum. All experiments followed the guidelines established by the Japanese Pharmacological Society and Institute for Experimental Animals at Meijo University. Protocols were approved by the Animal Ethics Board of Meijo University [permit no. (2017–2022)-11]. All efforts were made to minimize animal suffering and to reduce the number of animals used.

### 2.2. Drugs

Psilocin was synthesized within the Japanese law according to the methods published by Shirota et al. (Shirota et al., 2003), and its purity was verified by nuclear magnetic resonance analysis (Avance III 600, Bruker, MA, USA). (±)-1-(2,5-Dimethoxy-4-iodophenyl)-2-aminopropane (DOI), fluoxetine, and imipramine were obtained from Sigma-Aldrich (St. Louis, MO, USA). 4-bromo-3,6-dimethoxybenzocyclobuten-1-yl)methylamine hydrobromide (TCB-2) was obtained from Tocris Biosciences (Minneapolis, MN, USA). 6-chloro-2,3-dihydro-5-methyl-*N*-[6-[(2-methyl-3-pyridinyl)oxy]-3-pyridinyl]-1*H*-indole-1-carboxamide dihydrochloride (SB242084) and (*R*)-(2,3-dimethoxyphenyl)(1-(2-(4-fluorophenyl)ethyl)piperidin-4-yl methanol (volinanserin) were obtained from Cayman Chemical (Ann Arbor, MI, USA). Psilocin was dissolved to final doses of 1.5, 2.0, and 4.0 mg/kg in 0.9% NaCl solution (saline). DOI concentration was adjusted to 10 mg/mL using dimethyl sulfoxide (DMSO; Sigma-Aldrich) and dissolved in saline to obtain the final doses of 0.05, 0.1, 0.25, 1.0, and 2.0 mg/kg. The concentration of TCB-2 was adjusted to 10 mg/mL using DMSO and dissolved to final doses of 0.5, 1.0, and 5.0 mg/kg in saline. The concentration of fluoxetine was adjusted to 50 mg/mL using DMSO and dissolved to a final dose of 10 mg/kg using saline. SB242084 concentration was adjusted to 10 mg/mL using DMSO and dissolved to final doses of and 3.0 mg/kg using saline. Volinanserin was adjusted to 10 mg/mL using DMSO and dissolved in saline to a final dose of 1.0 mg/kg using saline. The imipramine dose was adjusted to 5.0 mg/kg using saline. All drugs were administered intraperitoneally (i.p.) at a dose of 10 mL/kg.

### 2.3. Forced-swimming test

Forced-swimming test (FST) was performed to measure depression-like behavior as described previously, with minor modifications (Takaba et al., 2022). Mice were individually placed in a clear beaker (19.3 cm in height, 13.1 cm in diameter; IWAKI, Tokyo, Japan) at a depth of 15 cm under water (25 ± 1°C) to ensure that mice could not touch the bottom of the beaker with their hind paws and tails and; allowed to swim freely for 15 min. Mice are generally immobile when they make no further attempts to escape except for the necessary movements to maintain their balance. All sessions were recorded using a video camera. The immobility time was measured in a double-blind manner.

### 2.4. Tail-suspension test

Tail-suspension test (TST) was carried out as described previously, with some modifications (Can et al., 2012). Mice were individually suspended in a tail suspension box (30 cm length × 30 cm width × 30 cm height; BrainScience Idea. Co., Ltd., Osaka, Japan) using a sellotape attached 1 cm from the end of the tail under moderate light conditions (20 lx) for 5 min. The immobility time was automatically recorded and analyzed using the Ethovision XT 12 automated tracking program (Noldus, Wageningen, Netherlands).

### 2.5. Novelty-suppressed feeding test

The novelty suppressed feeding test (NSFT) was performed to measure anxiety-like behavior by presenting regular chow to food-deprived mice (Elsayed et al., 2012). Mice were food-restricted for 24 h prior to the NSFT. A single food pellet was placed in the center of a bright light (100 lx) arena (31 cm long × 31 cm wide × 15 cm heigh) filled with wood chip bedding material. Each mouse was placed individually at the center of the apparatus and allowed to move freely in the cage for 10 min. The exploratory behaviors of all mice were recorded using a video camera. The latency to feed, feeding counts, and feeding time data of each mouse were collected in a double-blind manner.

### 2.6. Head-twitch response test

The head-twitch response (HTR) consists of rapid side-to-side head movements and is used to test hallucination-related behaviors in rodents (Halberstadt and Geyer, 2011). Mice were placed in an observation cage (29 cm long × 18 cm wide × 12 cm heigh) under moderate light conditions (15 lx) immediately or 24 h after drug administration. The HTR was recorded for 30 min using by a video camera. Drug-induced HTR (defined as rapid rotations of the head that can be distinguished from species-appropriate grooming or scratching behaviors) was counted as described previously, with minor modifications (Gonzalez-Maeso et al., 2007, Ibi et al., 2017) in a double-blind manner.

### 2.7. Three-chamber sociability test

A three-chamber sociability test was used to assess sociability and interest in social novelty and discrimination. This test was carried out as described previously, with some modifications (De Gregorio et al., 2021). The three-chamber apparatus (62 cm long × 41 cm wide × 22.5 cm heigh; BrainScience Idea. Co., Ltd.) consisted of three chambers (left, center, and right) measuring 21 cm long × 41 cm wide × 22.5 cm heigh, with walls separated by transparent acrylic. The walls to the center chamber had 4 cm width × 8 cm length cut-out doors, allowing movement between the chambers. Small wire cages (diameter, 10 cm) were placed in the right and left chambers, 4 cm from the walls. During habituation, the subject mouse was allowed to habituate to the apparatus and freely explore the three chambers for 15 min. An empty wire cage was placed in the right and left chambers under moderate light conditions (20 lx). During the sociability session, an unfamiliar male C57BL/6J mouse (stranger1) that had never met the subject mouse was placed in a wire cage in the left chamber, and an empty wire cage was placed in the right chamber. The subject mouse was introduced into the central chamber and allowed to freely explore the three chambers for 15 min. In the social novelty session, another unfamiliar male C57BL/6J mouse (stranger2) that had never met the subject mouse was placed in an empty wire cage in the right chamber, while the stranger1 remained in the cage in the left chamber. The subject mouse was introduced into the middle chamber and allowed to explore the three chambers freely for 15 min. After each session, the subject mouse was returned to its home cage for 10 min, and the three-chamber apparatus and wire cages were cleaned with purified water.

The time spent interacting with stranger1, stranger2, and the empty cage was automatically measured using the Ethovision XT 12 automated tracking program (Noldus). In order to reduce the variability among mice, the “sociability index” in each mouse was employed. This index was calculated using the following formula: [(interaction time with stranger1–interaction time with empty cage) / (interaction time with stranger1 + interaction time with empty cage) × 100]. The social novelty index was similarly calculated using the following formula: [(interaction time with stranger2– interaction time with stranger1)/(interaction time with stranger2 + interaction time with stranger1) × 100].

### 2.8. Locomotor activity

Locomotor activity was assessed as previously described (Ibi et al., 2020). Each mouse was placed in a standard transparent rectangular rodent cage (40 cm long × 45 cm wide × 26 cm high) under moderate light conditions (15 lx). The locomotor activity of each mouse was automatically measured for 15 min using digital counters with infrared sensors (Scanet SV-40; Melquest Co. Ltd., Toyama, Japan) 24 h after drug administration.

### 2.9. Statistical analysis

Prism 8 (GraphPad Software, Inc., San Diego, CA, USA) was used for statistical analyses, and figure generation. Since it was impossible to assume that the behavioral data had a Gaussian distribution, the data were expressed as medians and interquartile ranges. Significance was evaluated using the Kruskal–Wallis nonparametric one-way or two-way analysis of variance (ANOVA), followed by Bonferroni’s test for multiple comparisons. For the three-chamber sociability test, the interaction times with the cages in the left and right chambers within each group were statistically compared using the Wilcoxon matched-pairs signed-rank test in all sessions. A value of *p* < 0.05 was considered statistically significant.

## 3. Results

### 3.1. Effect of serotonergic psychedelic administrations on the immobility time in the FST in mice

**Figure 1.**
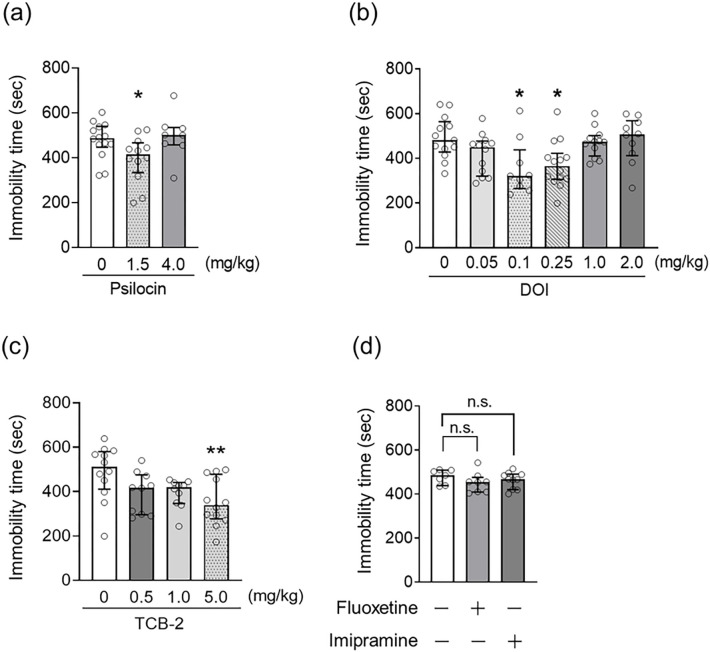
Effect of serotonergic psychedelic administrations on the immobility time in the FST in mice The FST was performed 24 h after drug administration in mice, and the immobility time was measured for 15 min (a-d). The doses of psilocin (a), DOI (b), and TCB-2 (c) were examined in the FST at the doses indicated below the vertical line of each graph. The effects of fluoxetine at 10 mg/kg and imipramine 5.0 mg/kg (d) were also examined. Values represent the median with interquartile range (n=7–14). Significance levels: *p<0.05, **p<0.01, vs. vehicle (Kruskal–Wallis nonparametric one-way ANOVA followed by Bonferroni’s test). n.s.: not significant.

To examine the dose-dependency of the antidepressant-like effects of serotonergic psychedelics, male C57BL/6J mice were subjected to the FST 24 h after administration of psilocin (1.5, 2.0, and 4.0 mg/kg), DOI (0.05, 0.1, 0.25, 1.0, and 2.0 mg/kg), or TCB-2 (0.5, 1.0, and 5.0 mg/kg). Mice that received psilocin (1.5 mg/kg), DOI (0.1 and 0.25 mg/kg), or TCB-2 (5.0 mg/kg) had significantly decreased immobility time in the FST compared with vehicle-treated control mice. Other tested doses of psilocin, DOI, and TCB-2 had no significant effect on the immobility time in the FST (Kruskal–Wallis nonparametric ANOVA followed by Bonferroni’s test; psilocin: H(3) = 6.80, *p* = 0.033, Fig. 1a; DOI: H(6)=16.06, *p* = 0.0067, Fig. 1b; TCB-2: H(4) = 8.26, *p* = 0.035, Fig. 1c). In contrast, acute treatment with the antidepressants fluoxetine, a selective serotonin reuptake inhibitor, and imipramine, a tricyclic antidepressant, did not affect immobility time in the FST 24 h after treatment (Kruskal–Wallis nonparametric ANOVA followed by Bonferroni’s test; H(3) = 2.73, *p* = 0.26, Fig. 1d). These results suggest that psychedelics have antidepressant-like effects at optimal concentration in mice. As the optimal concentrations, psilocin, DOI, and TCB-2 were henceforth used at 1.5 mg/kg, 0.1 mg/kg, and 5.0 mg/kg, respectively, unless otherwise indicated.

### 3.2. Role of 5-HT2A in the antidepressant-like effect of serotonergic psychedelics in mice

**Figure 2.**
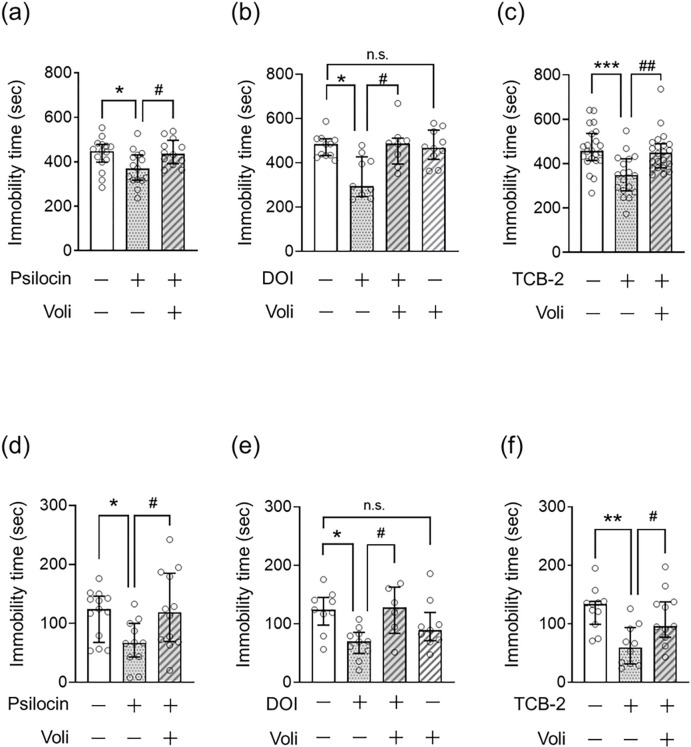
Role of 5-HT2A in the antidepressant-like effect of serotonergic psychedelics in mice FST (a-c) or TST (d-f) was carried out 24 h after administration of psilocin at 1.5 mg/kg (a, d), DOI at 0.1 mg/kg (b, e), or TCB-2 at 5.0 mg/kg (c, f) in mice, in which the immobility time was measured for 15 or 5 min, respectively. Volinanserin (Voli) at 1.0 mg/kg was injected 30 min before serotonergic psychedelic administration. Values represent the median with interquartile range (n=6–22). Significance levels: *p<0.05, **p<0.01, ***p<0.001 vs. vehicle; ^#^*p*<0.05, ^##^*p*<0.01, vs. serotonergic psychedelic alone (Kruskal–Wallis nonparametric one-way ANOVA followed by Bonferroni’s test). n.s.: not significant.

To confirm the involvement of 5-HT2A in the antidepressant effects of psilocin, DOI, and TCB-2, we assessed the effect of pretreatment with the 5-HT2A antagonist volinanserin on the decreased immobility time in the FST and TST in mice treated with psychedelics. As depicted in Fig. 1, psilocin (1.5 mg/kg), DOI (0.1 mg/kg), and TCB-2 (5.0 mg/kg) decreased the immobility time in the FST and TST, and pretreatment with volinanserin prevented this effect of serotonergic psychedelics in the FST (Kruskal–Wallis nonparametric ANOVA followed by Bonferroni’s test; psilocin: H(3) = 7.11, *p* = 0.029, Fig. 2a; DOI: H(4)=11.22, *p* = 0.011, Fig. 2b; TCB-2: H(3) = 15.68, *p* = 0.00040, Fig. 2c) and TST (Kruskal–Wallis nonparametric ANOVA followed by Bonferroni’s test; psilocin: H(3) = 7.27, *p* = 0.026, Fig. 2d; DOI: H(4)=9.95, *p* = 0.019, Fig. 2e; TCB-2: H(3) = 11.06, *p* = 0.0040, Fig. 2f).

Next, we investigated the contribution of 5-HT2C to the antidepressant-like effects of psilocin and DOI because serotonergic psychedelics have a modest affinity for 5-HT2C (Table 1). We tested the effect of pretreatment with the 5-HT2C antagonist SB242084 on the decreased immobility time in the FST of mice treated with psilocin or DOI.As shown in Figs. 1 and 2, the administration of psilocin or DOI significantly decreased the immobility time in the FST 24 h after treatment, which was not influenced by pretreatment with SB242084 (Kruskal–Wallis nonparametric ANOVA followed by Bonferroni’s test; psilocin: H(4) = 15.21, *p* = 0.0016, Supplemental Fig. 1a; DOI: H(3)=7.87, *p* = 0.020, Supplemental Fig. 1b), suggesting that serotonergic psychedelics have an antidepressant-like effect through 5-HT2A, rather than 5-HT2C.

To rule out the potential effect of volinanserin alone, mice were subjected to the FST and TST 24 h after the administration of a single dose of volinanserin. There was no significant difference in the immobility time in the FST (Fig. 2b) and TST (Fig. 2e) between vehicle-treated control and volinanserin-treated mice.

### 3.3. Role of 5-HT2A in the anxiolytic-like effect of serotonergic psychedelics in mice

**Figure 3.**
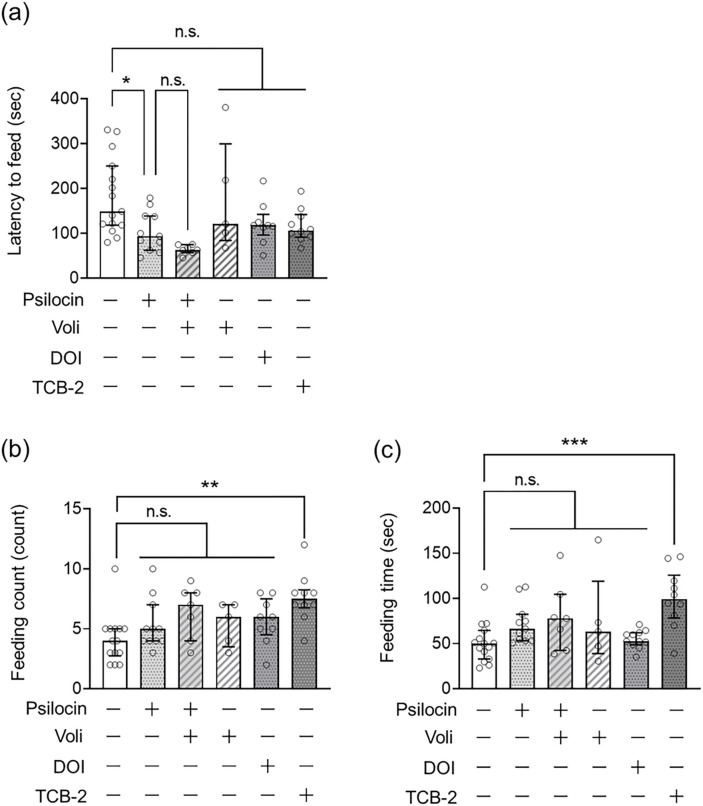
Role of 5-HT2A in the anxiolytic-like effect of serotonergic psychedelics in mice Mice were administrated serotonergic psychedelic (psilocin at 1.5 mg/kg, DOI at 0.1 mg/kg, or TCB-2 at 5.0 mg/kg) 24 h before the NSFT, and food was restricted for 24 h until the test. During this test, latency to feed (a), feeding count (b), and feeding time (c) were measured. Volinanserin (Voli) at 1.0 mg/kg was injected 30 min before psilocin administration. Values represent the median with interquartile range (n=5–15). Significance levels: *p<0.05, **p<0.01, ***p<0.001 vs. vehicle (Kruskal–Wallis nonparametric one-way ANOVA followed by Bonferroni’s test). n.s.: not significant.

Next, we examined the involvement of 5-HT2A in the anxiolytic-like effects of serotonergic psychedelics. The NSFT has been used to test anxiety-like behaviors in rodents. The latency to consume pellets was measured as an indicator of anxiety-like behavior in a novel environment after fasting for 24 h. Mice were subjected to the NSFT 24 h after treatment with psilocin, DOI, or TCB-2. Psilocin treatment significantly reduced the latency to feed (Fig. 3a), which was not attenuated by pretreatment with volinanserin (Kruskal–Wallis nonparametric ANOVA followed by Bonferroni’s test; H(6) = 21.70, *p* = 0.00090; Fig. 3a). In contrast, DOI or TCB-2 administration did not affect the latency to feed in the NSFT (Fig. 3a). These results suggest that psilocin exerts an anxiolytic effect independent of 5-HT2A.

Psilocin and DOI did not affect feeding count and time 24 h after psychedelic administration. However, mice treated with TCB-2 showed a significant increase in feeding count and time compared to vehicle-treated control mice (*p* < 0.001, Fig. 3b; *p* < 0.0001, Fig. 3c) (Kruskal–Wallis nonparametric ANOVA followed by Bonferroni’s test; feeding count: H(6) = 14.83, *p* = 0.011, Fig. 3b; feeding time: H(6) = 17.69, *p* = 0.0034, Fig. 3c). Further studies are needed to explain why TCB-2 affects feeding count and time in this test.

### 3.4. Effect of serotonergic psychedelic administrations on social behavior in the three-chamber sociability test in mice

**Figure 4.**
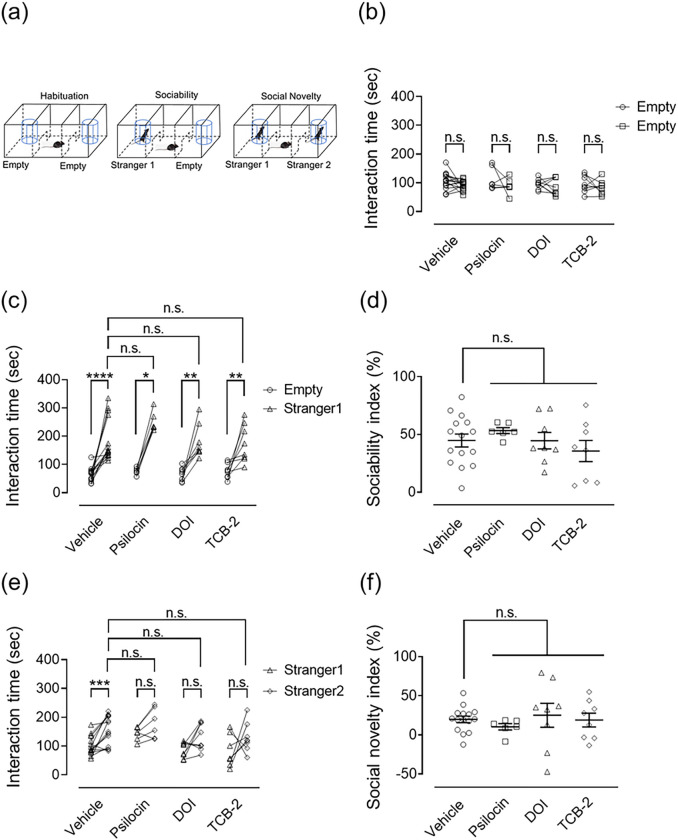
Effect of serotonergic psychedelic administrations on social behavior in the three-chamber sociability test in mice The mice were first habituated to the experimental apparatus 24 h after psychedelic administration, and then sociability and social novelty sessions were performed sequentially. All sessions were performed every 10 minutes for 15 minutes. Experimental depiction of the habituation (left), sociability (center), and social novelty (right) sessions (a). In the sociability session, one cage contained an unfamiliar mouse (stranger1), while the other was empty. The subject mouse was allowed to freely explore the three chambers. In the social novelty session, another unfamiliar mouse (stranger2) was placed in the remaining empty cage opposite stranger1, and the subject mouse was allowed to explore freely within the three chambers. First, the time spent interacting with both empty cages was measured (habituation b). Next, the time spent interacting with either stranger1 or the empty cage was measured (sociability sessions; c and d). Finally, the time spent interacting with stranger1 or stranger2 was measured (social novelty sessions; e and f). To reduce variability among mice, we calculated the sociability (d) and social novelty (f) indices. The Wilcoxon matched-pairs signed rank test was used to compare the interaction times between cages in each group. Kruskal–Wallis nonparametric one-way ANOVA followed by Bonferroni’s test was used to compare sociability and social novelty indices and the interaction time with strangers1 or 2 among groups. Values represent the median with interquartile range (n=6–15). Significance levels: *p<0.05, **p<0.01, ****p<0.0001, vs. empty (c), ***p<0.001 vs. stranger1 (e) (Wilcoxon matched-pairs signed rank test (b, c, e)). n.s.: not significant.

In this study, we tested the effects of a single dose of serotonergic psychedelics on social behavior using the three-chamber sociability test (Fig. 4a). The mice were subjected to the test 24 h after treatment with psilocin, DOI, or TCB-2. In habituation, there were no statistically significant differences in the interaction time between empty cages among the groups (Wilcoxon matched-pairs signed-rank test, vehicle: W=−42.00, *p* = 0.25; psilocin: W=−7.00, *p* = 0.56; DOI: W=−12.00, *p* = 0.46; TCB-2: W=−10.00, *p* = 0.47; Fig. 4b). In the sociability session, mice treated with either the vehicle or serotonergic psychedelics showed an increase in the interaction time with the stranger1 mouse over the empty cage (Wilcoxon matched-pairs signed-rank test, vehicle: W = 120.00, *p* < 0.0001; psilocin: W = 21.00, *p* < 0.05; DOI: W=36.00, *p* < 0.01; TCB-2: W = 36.00, *p* < 0.01, Fig. 4c); while, no significant difference was noted in the interaction time with stranger1 among all groups (Kruskal–Wallis nonparametric ANOVA followed by Bonferroni’s test; H(4) = 5.29, *p* = 0.15, Fig. 4c). Similarly, there was no difference in the sociability index among the four groups (Kruskal–Wallis nonparametric ANOVA followed by Bonferroni’s test; H(4) = 2.19, *p* = 0.53, Fig. 4d). These results suggest that mice treated with or without psychedelics showed the same level of sociability. In the social novelty session, mice treated with the vehicle spent more time interacting with the stranger2 mouse than with the stranger1 mouse, whereas mice treated with serotonergic psychedelics showed no statistical difference (Wilcoxon matched-pairs signed-rank test, vehicle: W = 112.0, *p* < 0.001; psilocin: W = 17.00, *p* = 0.0938; DOI: W=22.00, *p* = 0.15; TCB-2: W = 18.00, *p* = 0.25; Fig. 4e). On the other hand, no differences were observed in the interaction time with stranger2 (Kruskal–Wallis nonparametric ANOVA followed by Bonferroni’s test; H(4) = 4.62, *p* = 0.20, Fig. 4e) and in the social novelty index (Kruskal–Wallis nonparametric ANOVA followed by Bonferroni’s test, H(4) = 2.68, *p* = 0.44, Fig. 4f) among the four groups during the social novelty session, indicating that even though the vehicle-treated control mice exhibited social novelty/discrimination, the mice treated with psychedelics did not show such behavior. A single dose of psychedelic acid may have negatively affected social novelty/discrimination under the present experimental conditions.

### 3.5. Effect of serotonergic psychedelic administrations on the locomotor activities and the HTR in mice

**Figure 5.**
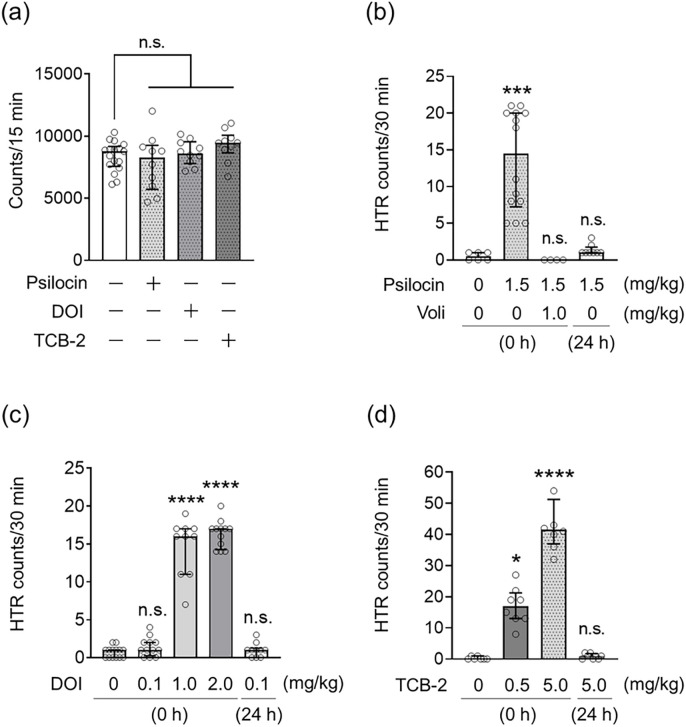
Effect of serotonergic psychedelic administrations on the locomotor activities and the HTR in mice Spontaneous locomotor activity was measured for 15 min 24 h after administration of psilocin at 1.5 mg/kg, DOI at 0.1 mg/kg, or TCB-2 at 5.0 mg/kg (a). HTR were counted for 30 min, either immediately or 24 h after administration of psilocin (b), DOI (c), and TCB-2 (d). Psychedelics dosages are indicated below the vertical line in each graph. Volinanserin (Voli) at 1.0 mg/kg was injected 30 min before psilocin administration. Values represent the median with interquartile range (n=4–17). Significance levels: *p<0.05, ***p<0.001, ****p<0.0001 vs. vehicle (Kruskal–Wallis nonparametric one-way ANOVA followed by Bonferroni’s test). n.s.: not significant.

Spontaneous locomotor activity in mice was measured for 15 min 24 h after psilocin, DOI, or TCB-2 administration, and no significant differences were found among the groups (Kruskal–Wallis nonparametric ANOVA followed by Bonferroni’s test; H(4) = 5.01, *p* = 0.17, Fig. 5a). Next, to assess the expression of hallucination-like behaviors in mice following the administration of serotonergic psychedelics, we investigated HTR for 30 min either immediately or 24 h after treatment with serotonergic psychedelics. Mice showed significantly greater HTR immediately after treatment with psilocin (1.5 mg/kg), DOI (1.0 or 2.0 mg/kg), or TCB-2 (5.0 mg/kg) (Fig. 5b–d). Interestingly, the mice showed no HTR induction even immediately after treatment with DOI (0.1 mg/kg) (Fig. 5c). On the other hand, psilocin (1.5 mg/kg), DOI (0.1 mg/kg), or TCB-2 (5.0 mg/kg) did not affect the HTR in mice 24 h after treatment (Kruskal–Wallis nonparametric ANOVA followed by Bonferroni’s test; psilocin: H(4) = 26.71, *p* < 0.0001, Fig. 5b; DOI: H(5)=42.50, *p* < 0.0001, Fig. 5c; TCB-2: H(4) = 26.60, *p* < 0.0001, Fig. 5d).

### 3.6. Long-lasting antidepressant-like effect of serotonergic psychedelics in the FST in mice

**Figure 6.**
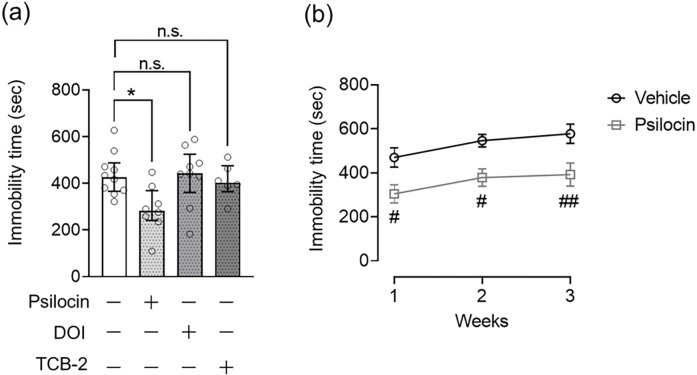
Long-lasting antidepressant-like effect of serotonergic psychedelics in the FST in mice FST was carried out in mice a week after administration of psilocin at 1.5 mg/kg, DOI at 0.1 mg/kg, or TCB-2 at 5.0 mg/kg, in which the immobility time was measured for 15 min (a). The FST was repeatedly performed every week for 3 weeks in mice treated with psilocin, and immobility time was measured for 15 min (b). Values represent the median with interquartile range (n=6–10). Significance levels: *p<0.05, vs. vehicle (Kruskal–Wallis nonparametric one-way ANOVA followed by Bonferroni’s test), ^#^*p*<0.05, ^##^*p*<0.01, vs. vehicle group at the corresponding time (two-way ANOVA followed by Bonferroni’s test). n.s.: not significant.

A previous clinical study showed that psilocybin treatment had a long-lasting effect on MDD for 12 months (Gukasyan et al., 2022). In the present study, we evaluated the persistent antidepressant-like effects of serotonergic psychedelics in mice. To accomplish this, the mice were administered psilocin, DOI, or TCB-2, and one week later, they were subjected to the FST. A decreasing effect of psilocin on immobility time was observed compared to that in vehicle-treated control mice. In contrast, DOI and TCB-2 did not affect the immobility time a week after treatment (Kruskal–Wallis nonparametric ANOVA followed by Bonferroni’s test; H(4) = 9.23, *p* = 0.026, Fig. 6a). To further evaluate how long this effect of psilocin persisted, the FST was repeated every week for a month. As a result, psilocin significantly and persistently decreased immobility time for three weeks after administration (two-way ANOVA followed by Bonferroni’s test; FTreatment(1,38) = 25.36, *p* < 0.0001; FTime(2,38) = 2.92, *p* = 0.067; FTreatment × Time(2,38) = 0.035, *p* = 0.96, Fig. 6b), but the effect was not observed four weeks after psilocin administration [Mann–Whitney U test, U = 15, *p* = 0.15, median with interquartile range, immobility time (sec); vehicle: 564.75 (263.31–647.03), psilocin: 462.99 (175.87–495.61); data not shown]. These results suggested that psilocin, but not DOI or TCB-2, may have long-lasting antidepressant effects.

## 4. Discussion

It is well known that 5-HT2A activation is necessary for psychedelic experiences (López-Giménez and González-Maeso, 2018), but the involvement of 5-HT2A in the therapeutic effects of psychedelics remains unknown. In a preclinical study, a single dose of psilocybin reportedly improved anhedonic behaviors in mice with chronic stress in the sucrose preference and female urine sniffing tests, which were not blocked by pretreatment with ketanserin, a 5-HT2A/2C antagonist (Hesselgrave et al., 2021). In contrast, pretreatment with ketanserin prevented the antidepressant-like effects of tabernanthalog, a non-hallucinogenic derivative of the psychedelic alkaloid ibogaine, in the FST of mice (Cameron et al., 2021). Based on these findings, the requirement of 5-HT2A for the therapeutic effects of serotonergic psychedelics is still controversial (Ripoll et al., 2006, Cameron et al., 2021, Cao et al., 2022). Considering our current study showing that volinanserin suppressed the antidepressant-like effects of serotonergic psychedelics, 5-HT2A activation by psychedelics may at least contribute to their antidepressant-like effects.

Regarding effects other than antidepressant effects, the present study showed an anxiolytic-like effect of psilocin, but not of DOI or TCB-2, in the NSFT of mice; however, the observed effect of psilocin was not attenuated by pretreatment with volinanserin (Fig. 3a), suggesting that psilocin induces anxiolytic effects independent of 5-HT2A receptors. In this context, DOI and TCB-2 may not have anxiolytic effects because they have a higher selectivity for 5-HT2A than psilocin. Meanwhile, psilocin shows moderate to high affinity for 5-HT2A, 5-HT1A, and 5-HT2C (Table 1) (Nichols, 2012). This raises the possibility that differences in the effects of psychedelics may be attributed to differences in their affinity for serotonergic receptors, including 5-HT2A.

In the present study, a single dose of psilocin, DOI, or TCB-2 did not affect the social behavior in naïve mice without stress in the three-chamber sociability test (Fig. 4). Interestingly, rodents with either social isolation in early life or chronic social defeat stress in adulthood reportedly display social dysfunction (Garcia-Pardo et al., 2015, Jianhua et al., 2017, Rivera-Irizarry et al., 2020), which can be prevented by SSRI or ketamine treatment (Scarborough et al., 2021, Ortiz et al., 2022). Meanwhile, SSRI and ketamine did not affect social function in naïve animals (Scarborough et al., 2021, Ortiz et al., 2022), which is consistent with the current study. This led us to consider whether the prosocial effects of acute psychedelic administration may become more evident in animal stress models. Therefore, future studies should test the prosocial effects of psychedelics in animal stress models.

Similar to previous reports (Halberstadt et al., 2011, Haberzettl et al., 2014, Klein et al., 2021), the current study demonstrated that hallucination-like behaviors occurred immediately after treatment with psilocin and TCB-2 in mice, although the effects were diminished 24 h after treatments (Fig. 5b, d). Additionally, DOI showed antidepressant-like effects in mice (Fig. 1b and 2b, e) but did not induce hallucination-like behavior immediately after administration, even at the same concentration (Fig. 5c). These results suggest that the underlying mechanisms of the antidepressant effect may be different from those of hallucinations, even though both effects are evoked by 5-HT2A stimulation. Thus, if the antidepressant effect could be differentiated from the hallucinatory effects, this could potentially promote the development of highly effective antidepressants that do not induce hallucinations. To realize this, we should discuss two possibilities: the molecular and neuronal discrimination of the antidepressant effect. First, the molecular aspects should be discussed. Since 5-HT2A is a Gαq protein-coupled receptor, it activates the Gq protein as a matter of course. On the other hand, signaling pathways of Gαi/o, Gα12/13, and β-arrestin, a scaffolding protein involved in receptor internalization and desensitization, are also activated by 5-HT2A stimulation (Pottie and Stove, 2022). A recent study with a newly created psychedelic analog suggested that the stimulation of the orthosteric binding pocket of 5-HT2A induces both hallucinatory and antidepressant effects via the Gq-mediated pathway, while stimulation of the allosteric pocket induces an antidepressant effect via the β-arrestin pathway without inducting hallucinations; that is, β-arrestin-biased 5-HT2A agonists may selectively lead to antidepressant effects (Cao et al., 2022, Yin and Gao, 2023). In the present study, DOI at 0.1 mg/kg showed an antidepressant-like effect without inducing HTR in mice, raising the possibility that DOI may activate the β-arrestin pathway rather than the Gq-related pathway, acting as a β-arrestin-biased agonist.

Second, the neuronal bases that isolate the antidepressant effects of psychedelics should be discussed. Previous studies have suggested that the activation of 5-HT2A in the cortex, such as the medial prefrontal and visual cortices, contributes to the hallucinatory-like effects of psychedelics in rodents (Willins and Meltzer, 1997, Gonzalez-Maeso et al., 2007, Michaiel et al., 2019). However, brain regions responsible for the antidepressant effects of psychedelic drugs remain unknown. If 5-HT2A activity in the cortex is not necessary for the antidepressant effect of psychedelics, it may be possible to separate the antidepressant effect from hallucinations by manipulating 5-HT2A activity in a brain region-specific manner. Taken together, the molecular and/or neuronal basis responsible for the antidepressant effects of serotonergic psychedelics should be elucidated to isolate the antidepressant effects of the drugs from the hallucinatory and other effects of psychedelics.

Surprisingly, psilocybin administration has shown sustained antidepressant effects for at least 12 months in patients with MDD (Gukasyan et al., 2022). However, the effect of ketamine lasts for only 1–2 weeks at most (Rong et al., 2018, Wilkinson et al., 2018). Similarly, a previous preclinical study showed that the antidepressant-like effects of LSD and psilocybin persisted for more than five weeks. In contrast, the effect of ketamine lasts for only one week in rats (Hibicke et al., 2020). The effects of psilocybin observed in rats are also supported by the present study. Taken together, the antidepressant-like effect of psilocybin (psilocin) appeared to last longer than that of ketamine. Moreover, a multicriteria decision analysis off various abused drugs in the United Kingdom found that LSD and magic mushrooms (psilocybin) are dependent and dissociate more than other drugs, such as ketamine, tobacco, and alcohol (Nutt et al., 2010). These results that serotonergic psychedelics such as psilocybin are superior to ketamine in terms of safety and efficacy.

## Figures and Tables

**Table 1:** Affinity values (Ki) of serotonergic psychedelics towards 5-HT1A, 5-HT2A, and 5-HT2C

5-HT receptor subtypes (Rats)				
Psychedelics	5-HT1A	5-HT2A	5-HT2C	References
Psilocin	49 nM	25 nM	10 nM	(Blair et al., 2000)
DOI	n.d.	0.79 nM	3.01 nM	(Knight et al., 2004)
TCB-2	n.d.	0.73 nM	n.d.	(McLean et al., 2006)

n.d.=not determined

## Data Availability

The datasets created during this investigation are available upon request from the corresponding author.

## References

[1] Ballard ED, Yarrington JS, Farmer CA, Richards E, Machado-Vieira R, Kadriu B, Niciu MJ, Yuan P, Park L, Zarate CA Jr. (2018) Characterizing the course of suicidal ideation response to ketamine. J Affect Disord 241: 86–93 30099268 10.1016/j.jad.2018.07.077 PMC6193483

[2] Berman RM, Cappiello A, Anand A, Oren DA, Heninger GR, Charney DS, Krystal JH (2000) Antidepressant effects of ketamine in depressed patients. Biol Psychiatry 47: 351–354 10686270 10.1016/s0006-3223(99)00230-9

[3] Blair JB, Kurrasch-Orbaugh D, Marona-Lewicka D, Cumbay MG, Watts VJ, Barker EL, Nichols DE (2000) Effect of ring fluorination on the pharmacology of hallucinogenic tryptamines. J Med Chem 43: 4701–4710 11101361 10.1021/jm000339w

[4] Cameron LP, Tombari RJ, Lu J, Pell AJ, Hurley ZQ, Ehinger Y, Vargas MV, McCarroll MN, Taylor JC, Myers-Turnbull D, Liu T, Yaghoobi B, Laskowski LJ, Anderson EI, Zhang G, Viswanathan J, Brown BM, Tjia M, Dunlap LE, Rabow ZT, Fiehn O, Wulff H, McCorvy JD, Lein PJ, Kokel D, Ron D, Peters J, Zuo Y, Olson DE (2021) A non-hallucinogenic psychedelic analogue with therapeutic potential. Nature 589: 474–479 33299186 10.1038/s41586-020-3008-z PMC7874389

[5] Can A, Dao DT, Terrillion CE, Piantadosi SC, Bhat S, Gould TD (2012) The tail suspension test. J Vis Exp: e3769 22315011 10.3791/3769 PMC3353516

[6] Cao D, Yu J, Wang H, Luo Z, Liu X, He L, Qi J, Fan L, Tang L, Chen Z, Li J, Cheng J, Wang S (2022) Structure-based discovery of nonhallucinogenic psychedelic analogs. Science 375: 403–411 35084960 10.1126/science.abl8615

[7] Carhart-Harris R, Giribaldi B, Watts R, Baker-Jones M, Murphy-Beiner A, Murphy R, Martell J, Blemings A, Erritzoe D, Nutt DJ (2021) Trial of Psilocybin versus Escitalopram for Depression. N Engl J Med 384: 1402–1411 33852780 10.1056/NEJMoa2032994

[8] Carhart-Harris RL, Bolstridge M, Day CMJ, Rucker J, Watts R, Erritzoe DE, Kaelen M, Giribaldi B, Bloomfield M, Pilling S, Rickard JA, Forbes B, Feilding A, Taylor D, Curran HV, Nutt DJ (2018) Psilocybin with psychological support for treatment-resistant depression: six-month follow-up. Psychopharmacology (Berl) 235: 399–408 29119217 10.1007/s00213-017-4771-x PMC5813086

[9] Carhart-Harris RL, Bolstridge M, Rucker J, Day CM, Erritzoe D, Kaelen M, Bloomfield M, Rickard JA, Forbes B, Feilding A, Taylor D, Pilling S, Curran VH, Nutt DJ (2016) Psilocybin with psychological support for treatment-resistant depression: an open-label feasibility study. Lancet Psychiatry 3: 619–627 27210031 10.1016/S2215-0366(16)30065-7

[10] Davis AK, Barrett FS, May DG, Cosimano MP, Sepeda ND, Johnson MW, Finan PH, Griffiths RR (2021) Effects of Psilocybin-Assisted Therapy on Major Depressive Disorder: A Randomized Clinical Trial. JAMA Psychiatry 78: 481–489 33146667 10.1001/jamapsychiatry.2020.3285 PMC7643046

[11] De Gregorio D, Popic J, Enns JP, Inserra A, Skalecka A, Markopoulos A, Posa L, Lopez-Canul M, Qianzi H, Lafferty CK, Britt JP, Comai S, Aguilar-Valles A, Sonenberg N, Gobbi G (2021) Lysergic acid diethylamide (LSD) promotes social behavior through mTORC1 in the excitatory neurotransmission. Proc Natl Acad Sci U S A 118 10.1073/pnas.2020705118 PMC7865169 33495318

[12] Elsayed M, Banasr M, Duric V, Fournier NM, Licznerski P, Duman RS (2012) Antidepressant effects of fibroblast growth factor-2 in behavioral and cellular models of depression. Biol Psychiatry 72: 258–265 22513055 10.1016/j.biopsych.2012.03.003 PMC3401338

[13] Foong AL, Grindrod KA, Patel T, Kellar J (2018) Demystifying serotonin syndrome (or serotonin toxicity). Can Fam Physician 64: 720–727 30315014 PMC6184959

[14] Garcia-Pardo MP, Blanco-Gandia MC, Valiente-Lluch M, Rodriguez-Arias M, Minarro J, Aguilar MA (2015) Long-term effects of repeated social stress on the conditioned place preference induced by MDMA in mice. Prog Neuropsychopharmacol Biol Psychiatry 63: 98–109 26093344 10.1016/j.pnpbp.2015.06.006

[15] Gonzalez-Maeso J, Weisstaub NV, Zhou M, Chan P, Ivic L, Ang R, Lira A, Bradley-Moore M, Ge Y, Zhou Q, Sealfon SC, Gingrich JA (2007) Hallucinogens recruit specific cortical 5-HT(2A) receptor-mediated signaling pathways to affect behavior. Neuron 53: 439–452 17270739 10.1016/j.neuron.2007.01.008

[16] Goodwin GM, Aaronson ST, Alvarez O, Arden PC, Baker A, Bennett JC, Bird C, Blom RE, Brennan C, Brusch D, Burke L, Campbell-Coker K, Carhart-Harris R, Cattell J, Daniel A, DeBattista C, Dunlop BW, Eisen K, Feifel D, Forbes M, Haumann HM, Hellerstein DJ, Hoppe AI, Husain MI, Jelen LA, Kamphuis J, Kawasaki J, Kelly JR, Key RE, Kishon R, Knatz Peck S, Knight G, Koolen MHB, Lean M, Licht RW, Maples-Keller JL, Mars J, Marwood L, McElhiney MC, Miller TL, Mirow A, Mistry S, Mletzko-Crowe T, Modlin LN, Nielsen RE, Nielson EM, Offerhaus SR, O’Keane V, Palenicek T, Printz D, Rademaker MC, van Reemst A, Reinholdt F, Repantis D, Rucker J, Rudow S, Ruffell S, Rush AJ, Schoevers RA, Seynaeve M, Shao S, Soares JC, Somers M, Stansfield SC, Sterling D, Strockis A, Tsai J, Visser L, Wahba M, Williams S, Young AH, Ywema P, Zisook S, Malievskaia E (2022) Single-Dose Psilocybin for a Treatment-Resistant Episode of Major Depression. N Engl J Med 387: 1637–1648 36322843 10.1056/NEJMoa2206443

[17] Gukasyan N, Davis AK, Barrett FS, Cosimano MP, Sepeda ND, Johnson MW, Griffiths RR (2022) Efficacy and safety of psilocybin-assisted treatment for major depressive disorder: Prospective 12-month follow-up. J Psychopharmacol 36: 151–158 35166158 10.1177/02698811211073759 PMC8864328

[18] Haberzettl R, Fink H, Bert B (2014) Role of 5-HT(1A)- and 5-HT(2A) receptors for the murine model of the serotonin syndrome. J Pharmacol Toxicol Methods 70: 129–133 25087754 10.1016/j.vascn.2014.07.003

[19] Halberstadt AL, Geyer MA (2011) Multiple receptors contribute to the behavioral effects of indoleamine hallucinogens. Neuropharmacology 61: 364–381 21256140 10.1016/j.neuropharm.2011.01.017 PMC3110631

[20] Halberstadt AL, Koedood L, Powell SB, Geyer MA (2011) Differential contributions of serotonin receptors to the behavioral effects of indoleamine hallucinogens in mice. J Psychopharmacol 25: 1548–1561 21148021 10.1177/0269881110388326 PMC3531560

[21] Hesselgrave N, Troppoli TA, Wulff AB, Cole AB, Thompson SM (2021) Harnessing psilocybin: antidepressant-like behavioral and synaptic actions of psilocybin are independent of 5-HT2R activation in mice. Proc Natl Acad Sci U S A 118 10.1073/pnas.2022489118 PMC8092378 33850049

[22] Hibicke M, Landry AN, Kramer HM, Talman ZK, Nichols CD (2020) Psychedelics, but Not Ketamine, Produce Persistent Antidepressant-like Effects in a Rodent Experimental System for the Study of Depression. ACS Chem Neurosci 11: 864–871 32133835 10.1021/acschemneuro.9b00493

[23] Hofmann A, Heim R, Brack A, Kobel H (1958) [Psilocybin, a psychotropic substance from the Mexican mushroom Psilicybe mexicana Heim]. Experientia 14: 107–109 13537892 10.1007/BF02159243

[24] Ibi D, de la Fuente Revenga M, Kezunovic N, Muguruza C, Saunders JM, Gaitonde SA, Moreno JL, Ijaz MK, Santosh V, Kozlenkov A, Holloway T, Seto J, Garcia-Bea A, Kurita M, Mosley GE, Jiang Y, Christoffel DJ, Callado LF, Russo SJ, Dracheva S, Lopez-Gimenez JF, Ge Y, Escalante CR, Meana JJ, Akbarian S, Huntley GW, Gonzalez-Maeso J (2017) Antipsychotic-induced Hdac2 transcription via NF-kappaB leads to synaptic and cognitive side effects. Nat Neurosci 20: 1247–1259 28783139 10.1038/nn.4616 PMC5675106

[25] Ibi D, Nakasai G, Koide N, Sawahata M, Kohno T, Takaba R, Nagai T, Hattori M, Nabeshima T, Yamada K, Hiramatsu M (2020) Reelin Supplementation Into the Hippocampus Rescues Abnormal Behavior in a Mouse Model of Neurodevelopmental Disorders. Front Cell Neurosci 14: 285 32982694 10.3389/fncel.2020.00285 PMC7492784

[26] Jianhua F, Wei W, Xiaomei L, Shao-Hui W (2017) Chronic social defeat stress leads to changes of behaviour and memory-associated proteins of young mice. Behav Brain Res 316: 136–144 27609644 10.1016/j.bbr.2016.09.011

[27] Klein AK, Chatha M, Laskowski LJ, Anderson EI, Brandt SD, Chapman SJ, McCorvy JD, Halberstadt AL (2021) Investigation of the Structure-Activity Relationships of Psilocybin Analogues. ACS Pharmacol Transl Sci 4: 533–542 33860183 10.1021/acsptsci.0c00176 PMC8033608

[28] Knight AR, Misra A, Quirk K, Benwell K, Revell D, Kennett G, Bickerdike M (2004) Pharmacological characterisation of the agonist radioligand binding site of 5-HT(2A), 5-HT(2B) and 5-HT(2C) receptors. Naunyn Schmiedebergs Arch Pharmacol 370: 114–123 15322733 10.1007/s00210-004-0951-4

[29] López-Giménez JF, González-Maeso J (2018) Hallucinogens and Serotonin 5-HT(2A) Receptor-Mediated Signaling Pathways. Curr Top Behav Neurosci 36: 45–73 28677096 10.1007/7854_2017_478 PMC5756147

[30] McLean TH, Parrish JC, Braden MR, Marona-Lewicka D, Gallardo-Godoy A, Nichols DE (2006) 1-Aminomethylbenzocycloalkanes: conformationally restricted hallucinogenic phenethylamine analogues as functionally selective 5-HT2A receptor agonists. J Med Chem 49: 5794–5803 16970404 10.1021/jm060656o

[31] Michaiel AM, Parker PRL, Niell CM (2019) A Hallucinogenic Serotonin-2A Receptor Agonist Reduces Visual Response Gain and Alters Temporal Dynamics in Mouse V1. Cell Rep 26: 3475–3483 e3474 30917304 10.1016/j.celrep.2019.02.104 PMC6559379

[32] Nelsen MR, Dunner DL (1995) Clinical and differential diagnostic aspects of treatment-resistant depression. J Psychiatr Res 29: 43–50 7629754 10.1016/0022-3956(94)00042-p

[33] Nichols DE (2012) Structure-activity relationships of serotonin 5-HT2Aagonists. Wiley Interdisciplinary Reviews: Membrane Transport and Signaling 1: 559–579

[34] Nichols DE (2016) Psychedelics. Pharmacol Rev 68: 264–355 26841800 10.1124/pr.115.011478 PMC4813425

[35] Nichols DE (2018) Chemistry and Structure-Activity Relationships of Psychedelics. Curr Top Behav Neurosci 36: 1–43 10.1007/7854_2017_475 28401524

[36] Nutt D, Erritzoe D, Carhart-Harris R (2020) Psychedelic Psychiatry’s Brave New World. Cell 181: 24–28 32243793 10.1016/j.cell.2020.03.020

[37] Nutt DJ, King LA, Phillips LD, Independent Scientific Committee on D (2010) Drug harms in the UK: a multicriteria decision analysis. Lancet 376: 1558–1565 21036393 10.1016/S0140-6736(10)61462-6

[38] Odland AU, Kristensen JL, Andreasen JT (2021) Investigating the role of 5-HT2A and 5-HT2C receptor activation in the effects of psilocybin, DOI, and citalopram on marble burying in mice. Behav Brain Res 401: 113093 33359368 10.1016/j.bbr.2020.113093

[39] Ortiz V, Costa Campos R, Fofo H, Fernandez SP, Barik J (2022) Nicotinic receptors promote susceptibility to social stress in female mice linked with neuroadaptations within VTA dopamine neurons. Neuropsychopharmacology 47: 1587–1596 35459925 10.1038/s41386-022-01314-4 PMC9283477

[40] Pottie E, Stove CP (2022) In vitro assays for the functional characterization of (psychedelic) substances at the serotonin receptor 5-HT(2A) R. J Neurochem 162: 39–59 34978711 10.1111/jnc.15570

[41] Ripoll N, Hascoet M, Bourin M (2006) Implication of 5-HT2A subtype receptors in DOI activity in the four-plates test-retest paradigm in mice. Behav Brain Res 166: 131–139 16154212 10.1016/j.bbr.2005.07.013

[42] Rivera-Irizarry JK, Skelly MJ, Pleil KE (2020) Social Isolation Stress in Adolescence, but not Adulthood, Produces Hypersocial Behavior in Adult Male and Female C57BL/6J Mice. Front Behav Neurosci 14: 129 32792924 10.3389/fnbeh.2020.00129 PMC7394086

[43] Rong C, Park C, Rosenblat JD, Subramaniapillai M, Zuckerman H, Fus D, Lee YL, Pan Z, Brietzke E, Mansur RB, Cha DS, Lui LMW, McIntyre RS (2018) Predictors of Response to Ketamine in Treatment Resistant Major Depressive Disorder and Bipolar Disorder. Int J Environ Res Public Health 15 10.3390/ijerph15040771 PMC5923813 29673146

[44] Sapkota A, Khurshid H, Qureshi IA, Jahan N, Went TR, Sultan W, Alfonso M (2021) Efficacy and Safety of Intranasal Esketamine in Treatment-Resistant Depression in Adults: A Systematic Review. Cureus 13: e17352 34447651 10.7759/cureus.17352 PMC8381465

[45] Scarborough J, Mueller FS, Weber-Stadlbauer U, Mattei D, Opitz L, Cattaneo A, Richetto J (2021) A novel murine model to study the impact of maternal depression and antidepressant treatment on biobehavioral functions in the offspring. Mol Psychiatry 26: 6756–6772 34002019 10.1038/s41380-021-01145-7 PMC8760069

[46] Shirota O, Hakamata W, Goda Y (2003) Concise large-scale synthesis of psilocin and psilocybin, principal hallucinogenic constituents of “magic mushroom”. J Nat Prod 66: 885–887 12828485 10.1021/np030059u

[47] Singh JB, Daly EJ, Mathews M, Fedgchin M, Popova V, Hough D, Drevets WC (2020) Approval of esketamine for treatment-resistant depression. Lancet Psychiatry 7: 232–235 32087801 10.1016/S2215-0366(19)30533-4

[48] Takaba R, Ibi D, Watanabe K, Hayakawa K, Nakasai G, Hiramatsu M (2022) Role of sirtuin1 in impairments of emotion-related behaviors in mice with chronic mild unpredictable stress during adolescence. Physiol Behav 257: 113971 36183852 10.1016/j.physbeh.2022.113971

[49] Turner EH (2019) Esketamine for treatment-resistant depression: seven concerns about efficacy and FDA approval. Lancet Psychiatry 6: 977–979 31680014 10.1016/S2215-0366(19)30394-3

[50] Wilkinson ST, Ballard ED, Bloch MH, Mathew SJ, Murrough JW, Feder A, Sos P, Wang G, Zarate CA Jr, Sanacora G (2018) The Effect of a Single Dose of Intravenous Ketamine on Suicidal Ideation: A Systematic Review and Individual Participant Data Meta-Analysis. Am J Psychiatry 175: 150–158 28969441 10.1176/appi.ajp.2017.17040472 PMC5794524

[51] Willins DL, Meltzer HY (1997) Direct injection of 5-HT2A receptor agonists into the medial prefrontal cortex produces a head-twitch response in rats. J Pharmacol Exp Ther 282: 699–706 9262333

[52] Wisniewski SR, Rush AJ, Nierenberg AA, Gaynes BN, Warden D, Luther JF, McGrath PJ, Lavori PW, Thase ME, Fava M, Trivedi MH (2009) Can phase III trial results of antidepressant medications be generalized to clinical practice? A STAR*D report. Am J Psychiatry 166: 599–607 19339358 10.1176/appi.ajp.2008.08071027

[53] Yin YN, Gao TM (2023) Non-hallucinogenic Psychedelic Analog Design: A Promising Direction for Depression Treatment. Neurosci Bull 39: 170–172 35927548 10.1007/s12264-022-00933-7 PMC9849505

[54] Zhang K, Hashimoto K (2019) An update on ketamine and its two enantiomers as rapid-acting antidepressants. Expert Rev Neurother 19: 83–92 30513009 10.1080/14737175.2019.1554434

